# Experimental evidence indicating that mastreviruses probably did not co-diverge with their hosts

**DOI:** 10.1186/1743-422X-6-104

**Published:** 2009-07-16

**Authors:** Gordon W Harkins, Wayne Delport, Siobain Duffy, Natasha Wood, Adérito L Monjane, Betty E Owor, Lara Donaldson, Salem Saumtally, Guy Triton, Rob W Briddon, Dionne N Shepherd, Edward P Rybicki, Darren P Martin, Arvind Varsani

**Affiliations:** 1 South African National Bioinformatics Institute, University of the Western Cape, Cape Town, South Africa; 2 Institute of Infectious Disease and Molecular Medicine, University of Cape Town, Rondebosch, Cape Town, South Africa; 3 Antiviral Research Centre, Department of Pathology, University of California, San Diego, San Diego, 92103, USA; 4 Department of Ecology, Evolution and Natural Resources, Rutgers University, New Brunswick, NJ 08901, USA; 5 Centre for High-Performance Computing, Rosebank, Cape Town, South Africa; 6 Department of Molecular and Cell Biology, University of Cape Town, Rondebosch, Cape Town, 7701, South Africa; 7 Mauritian Sugar Industry Research Institute, Réduit, Mauritius; 8 Department of Disease and Stress Biology, John Innes Centre, Norwich NR4 7UH, UK; 9 National Institute for Biotechnology and Genetic Engineering, Jhang Road, P.O. Box 577, Faisalabad, Pakistan; 10 Electron Microscope Unit, University of Cape Town, Private Bag, Rondebosch 7701, South Africa; 11 School of Biological Sciences, University of Canterbury, Private Bag 4800, Christchurch, New Zealand

## Abstract

**Background:**

Despite the demonstration that geminiviruses, like many other single stranded DNA viruses, are evolving at rates similar to those of RNA viruses, a recent study has suggested that grass-infecting species in the genus *Mastrevirus *may have co-diverged with their hosts over millions of years. This "co-divergence hypothesis" requires that long-term mastrevirus substitution rates be at least 100,000-fold lower than their basal mutation rates and 10,000-fold lower than their observable short-term substitution rates. The credibility of this hypothesis, therefore, hinges on the testable claim that negative selection during mastrevirus evolution is so potent that it effectively purges 99.999% of all mutations that occur.

**Results:**

We have conducted long-term evolution experiments lasting between 6 and 32 years, where we have determined substitution rates of between 2 and 3 × 10^-4 ^substitutions/site/year for the mastreviruses Maize streak virus (MSV) and Sugarcane streak Réunion virus (SSRV). We further show that mutation biases are similar for different geminivirus genera, suggesting that mutational processes that drive high basal mutation rates are conserved across the family. Rather than displaying signs of extremely severe negative selection as implied by the co-divergence hypothesis, our evolution experiments indicate that MSV and SSRV are predominantly evolving under neutral genetic drift.

**Conclusion:**

The absence of strong negative selection signals within our evolution experiments and the uniformly high geminivirus substitution rates that we and others have reported suggest that mastreviruses cannot have co-diverged with their hosts.

## Background

It is becoming increasingly apparent that single-stranded DNA (ssDNA) viruses such as the anelloviruses [1-3], geminiviruses [4-9], parvoviruses [10-12] and microviruses [13,14] are probably evolving as rapidly as many RNA viruses [15]. While the inherent infidelities of RNA polymerases and reverse transcriptases drive the high rates of evolution seen in RNA viruses, all known ssDNA viruses replicate using presumably high-fidelity host DNA polymerases. It is surprising, therefore, that the basal mutation rates of ssDNA viruses are orders of magnitude higher than those of their hosts [15].

The best supported, non-exclusive theories that have so far been put forward to explain discrepancies between basal mutation rates of ssDNA viruses and their hosts are that: (1) when in a ssDNA state the genomes of these viruses are subject to mutagenic processes that are less frequently experienced in dsDNA [4]; (2) geminivirus genomes, and those of some other ssDNA viruses, are not sufficiently methylated such that normal host mechanisms of mismatch repair may not function during their replication [16,17]; and (3) when replicating, ssDNA virus genomes are only transiently double stranded such that when errors occur they are not efficiently repaired by host base-excision pathways [4].

Evidence is mounting that the rapid evolution of geminiviruses is, at least in part, driven by mutational processes that act specifically on ssDNA. Controlled evolution experiments involving Maize streak virus (MSV), a geminivirus in the *Mastrevirus *genus, have revealed a strand specific G → T mutation bias that is possibly attributable to oxidative damage to guanines [9]. Similarly, analyses of nucleotide substitution biases in natural tomato and cassava infecting geminivirus isolates (in the *Begomovirus *genus) have, in addition to similar G → T mutation biases, identified overrepresentations of C → T and G → A transitions. These biases indicate that geminivirus DNA may experience elevated rates of spontaneous damage while in a single stranded state [4,5]. Although it remains to be determined in a larger scale study whether an excess of C → T and G → A transitions have occurred during mastrevirus evolution, all these studies are consistent with the hypothesis that viral ssDNA is subjected to greater oxidative stresses (such as oxidative deamination of guanine and cytosine or oxidation of guanine to 8-oxoguanine) compared to host dsDNA.

High geminivirus basal mutation rates do not, however, necessarily imply that these viruses are also evolving rapidly. Rather than simply being the rate at which mutations occur, evolutionary rates are also influenced by (1) the rate at which deleterious mutations are purged from a population by negative, or purifying, selection, (2) the efficiency with which advantageous adaptive mutations are fixed in a population by positive, or diversifying, selection and (3) the rate at which neutral mutations (i.e. those mutations with no effect on fitness) are fixed in or lost from a population by random genetic drift. Adopting the convention of Duffy *et al*. [15] we differentiate between the biochemical or basal rate at which mutations arise (mutation rate, measured in rounds of genomic replication or units of time), and the usually slower rate at which mutations accumulate in wild populations evolving under natural selection (substitution rate, usually measured in years).

Geminiviruses have either one (monopartite, species in the *Begomovirus, Mastrevirus, Topocuvirus and Curtovirus *genera) or two (bipartite, species in the *Begomovirus *genus) ~2.7 Kb genome components. These compact genomes are among the smallest of any known viruses and encode only a small number of usually multifunctional and often overlapping genes [18]. Mastreviruses such as MSV and Wheat dwarf virus (WDV), for example, express only four distinct proteins: a movement protein (MP), a coat protein (CP), a replication associated protein (Rep) and a RepA protein, expressed from an alternative spliceform of the *rep *gene transcript such that it shares ~70% of its amino acid sequence with Rep [18]. The compactness of mastrevirus genomes is further emphasised by the fact that, with the exception of MP, these proteins have multiple known functions [18]. Given that many, if not most, mutations that occur in such compact genomes will be at least slightly deleterious and therefore subject to negative selection, it is expected that mastrevirus nucleotide substitution rates will be at least slightly lower than their basal mutation rates.

It is currently a matter of dispute as to how much lower geminivirus substitution rates are relative to their basal mutation rates. Experimental analyses of highly adaptive point mutations [19-21] and mutation frequencies in genomes sampled after 30–60 days of replication within infected plants [6,8,22] imply that the basal mutation rates of geminiviruses are in excess of 10^-3 ^mutations per site per year (mut/site/year). Correspondence between the phylogenies of certain mastrevirus species and those of their grass hosts has, however, prompted speculation that mastreviruses may have co-diverged with grasses and that their substitution rates may therefore be as low as 10^-8 ^substitutions per site per year (subs/site/year; [23]) – *i.e*. ten thousand times lower than their basal mutation rates. It is possible that very short-term evolution experiments (<0.2 years) produce inflated estimates of long-term substitution rates, because they are measuring adaptation (positive selection) to a novel host (*e.g*., [6,9]), or have not allowed sufficient time for negative selection to have effectively purged mildly deleterious mutations [24]. However, the co-divergence hypothesis demands a long-term substitution rate four orders of magnitude lower than the approximately 2 × 10^-4 ^to 7 × 10^-4 ^subs/site/year rates that have been estimated in short-term (<5 years) evolution experiments [7,9] and longer term (over tens of years) substitution rates estimated from temporally structured tomato and cassava infecting begomovirus datasets sampled from nature [4,5].

The ten-thousand-fold discrepancy between directly-calculated geminivirus substitution rate estimates and those implied by the co-divergence hypothesis is difficult to reconcile. It has been suggested that different evolutionary forces are operating over short- (less than one year), long- (tens of years) and very long-term (thousands of years) evolutionary timescales: even though point mutations rapidly accumulate in geminiviruses over observable timescales, over the millennia mastreviruses experience an almost complete absence of positive selection and neutral genetic drift, coupled with almost unfalteringly efficient negative selection [23]. This argument relies on the strange circumstance of mastrevirus species having had long co-evolutionary histories within their hosts, but without their having engaged in arms races with those hosts.

Here we describe a series of evolution experiments involving MSV and Sugarcane streak Réunion virus (SSRV – a mastrevirus species closely related to MSV [25]) that lasted between 6 and 32 years. Our results provide extensive additional support for the hypothesis that, as with other geminiviruses, MSV and SSRV basal mutation rates are possibly elevated by unrepaired oxidative damage inflicted on ssDNA. We additionally show that, contrary to expectations under the co-divergence hypothesis, neutral genetic drift and not negative selection appears to be a dominant process determining the fate of new mutations.

## Results and discussion

### Long term mastrevirus evolution experiments

In 1971, a sugarcane plant presenting with foliar streak symptoms later attributed to SSRV [25] was collected in Mauritius. In 1976, viruses were leafhopper transmitted from this plant to both a plant of the sugarcane variety H44-3098 and the wild grass species *Coix lachryma-jobi*. Both sugarcane and *Coix *plants were maintained in an insect free glasshouse over the next 32 years at the Mauritius Sugar Industry Research Institute. At some time between 1977 and 1986 viruses were retransmitted by leafhopper from the *Coix *to sugarcane, and in 1987 leaf samples from this sugarcane plant were shipped to Institut de Biologie Moleculaire et Cellulaire du CNRS in France, where total DNA was extracted and stored until 2008. In 1984, two stalks cut from the H44-3098 plant were sent to the John Innes Centre in the United Kingdom where they were planted and maintained until 1997. Total DNA was extracted from one of these plants in 1991, and symptomatic leaves from the other were cut in 1997 and stored at -80°C until DNA was extracted from them in 2007. In 1989, leaf samples from the H44-3098 plant were also shipped to the University of Cape Town in South Africa where total DNA was extracted and stored until 2008. Finally, in 2008 we obtained total leaf DNA samples from the originally infected *Coix *and H44-3098 plants in Mauritius.

In an unrelated experiment, two naturally-infected perennial *Digitaria *sp grasses with mild streak symptoms (later attributed to the MSV-strains MSV-B and MSV-F in each plant, respectively [26]) were maintained under insect-free conditions at the John Innes Centre in the United Kingdom between 1984 and 1997 [27]. Total genomic DNA was isolated and stored from each of these plants in 1991 and again in 1997.

**Figure 1:**
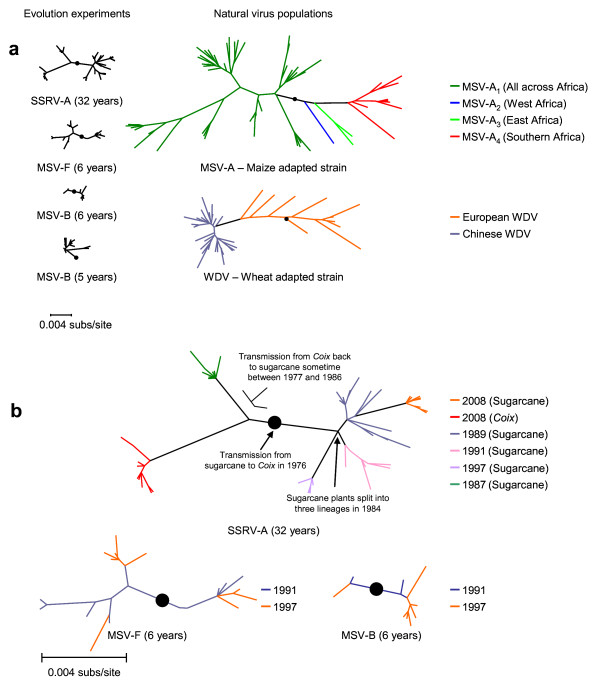
**Description of datasets**. (**a**) Phylogenetic comparison of sequences from experimental evolution experiments (left) and sequences sampled from nature (right), all drawn to the same scale. Whereas the SSRV-A (32 years), MSV-F (6 years) and MSV-B (6 years) datasets are described here for the first time, the MSV-B (5 years), MSV-A, and WDV datasets are those described by van der Walt *et al*. [9], Varsani *et al*. [26] and Ramsel *et al*. [28], respectively. Black dots indicate likely rooting positions as determined by an outgroup. Best fit models used during maximum likelihood tree construction are GTR+I+Γ_4 _for the SSRV, WDV and MSV-A trees, F81+Γ_4 _for the MSV-B five-year and MSV-F six-year trees and TN93+Γ_4 _for the MSV-B six-year tree. (**b**) Evolution experiment datasets indicating the sources and timing of sequence sampling.

To assess sequence divergence over time in these three serendipitous evolution experiments, we cloned and sequenced between 8 and 20 complete viral genomes from each of the six SSRV samples (a total of 81 clones), the two MSV-B samples (a total of 18 clones) and the two MSV-F samples (a total of 22 clones; see Table 1 for a breakdown of samples from which clones were obtained). We found that the viral diversity within the various experimental plants over the duration of the experiment was surprisingly high when compared with that observed within natural continent-wide MSV and WDV populations (Figure 1a). For example, the degree of virus diversification noted over the 32-year SSRV experiment is approximately (1) half that found for the major southern African MSV-A variant [26], MSV-A_4_, and (2) equivalent to that found throughout China for the wheat-adapted WDV strain [28].

**Table 1:** Breakdown of full genome sequences sampled during three separate evolution experiments and the results of neutrality tests indicate no significant deviation from neutral evolution in any of the samples.

				Neutrality tests^*a*^	
				
Experiment	Sample	Sequences	Variable sites	Tajima's D	Fu and Li's F*
32-year SSRV	All SSRV	81	125	-0.85	-2.01
	1987	9	13	-1.22	-1.20
	1989	20	34	-1.23	-1.31
	1991	10	15	-0.80	-1.12
	1997	11	7	-1.22	-1.38
	2008 (sugarcane)	12	12	-1.34	-1.73
	2008 (*Coix*)	19	14	-1.5	-1.33

6-year MSV-B	All MSV-B	18	26	-0.31	-1.10
	1991	10	11	-0.69	-0.10
	1997	8	23	-1.35	-1.55

6-Year MSV-F	All MSV-F	22	51	-0.42	-1.01
	1991	11	33	0.36	0.08
	1997	11	34	0.211	-0.13

^a ^All p-values are > 0.1 (i.e. there is no significant deviation from neutrality) for all tests other than for Fu and Li's F* with the full SSRV dataset which has a p-value between 0.05 and 0.1.

The amount of genetic variability observed in the two six-year-long experiments involving MSV-F and MSV-B in *Digitaria *spanned that previously observed in a five- year experiment involving MSV-B in sugarcane [9]. It was immediately apparent, however, that the virus population within the MSV-B infected plant was substantially less diverse over the course of the experiment than that within the MSV-F infected plant (Figure 1b).

It is important to point out that none of the three evolution experiments was initiated using cloned viruses and that we have no samples that were taken within two years of the start of the experiments. Therefore, the diverse virus populations within the infected plants could have arisen through rapid evolutionary rates, or as a result of the plants having been co-infected with divergent virus lineages – a situation that may have resulted in lineage sorting or founder effects.

However, when we compared the phylogenetic relationships of virus genomes sampled at consecutive time-points from individual plants (represented by blue and orange coloured branches on the trees in Figure 1b), we noted that samples from later time-points (orange branches in Figure 1b) were generally situated further from the presumed root-nodes than were those sampled at earlier time-points (blue branches in Figure 1b). Such a temporally-structured phylogenetic pattern indicated that, despite our knowing neither the precise genotypes of the viruses that initiated our experimental populations, nor the exact time of infection, we should still be able to accurately infer nucleotide substitution rates from our data.

### Geminiviruses have uniformly high nucleotide substitution rates

**Figure 2:**
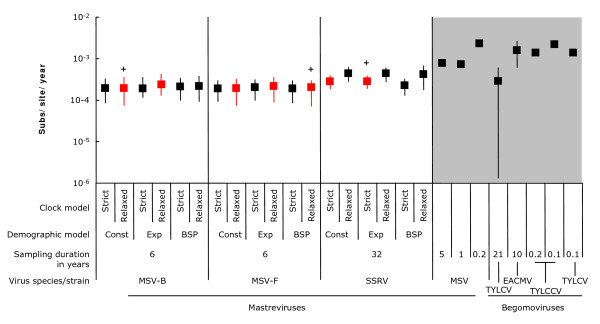
**The mean substitution rate estimates for MSV and SSRV are between 2.0 × 10^-4 ^and 3.5 × 10^-4 ^subs/site/year**. For the six-year MSV-B and MSV-F and the 32-year SSRV evolution experiments, substitution rate estimates made using a range of demographic and molecular clock models are presented. Whereas black squares indicate the most probable substitution rates, vertical bars indicate the 95% highest probability density of the substitution rate estimates. Red squares indicate rates estimated using the best fit demographic and clock models (determined using Bayes factor tests; Additional file 1). Stars indicates the models that returned the highest likelihood. When more than one red square is shown for a particular dataset this indicates that neither demographic model provided better support for the data. For purposes of comparison, previous estimates of substitution rates are presented (in the grey area) for both MSV (full genome sequences sampled during shorter term evolution experiments lasting between 2 months and 5 years; [9,22] from individual plants) and the begomoviruses, TYLCV (full genome sequences sampled from nature over 19 years [4]), East African cassava mosaic virus (EACMV, full genome sequences sampled from nature over 8 years [5]), Tomato yellow leaf curl China virus (TYLCCV, partial genome sequences sampled over 1 to 2 months from individual plants [6]) and TYLCV (full genome sequences sampled over 1 month from individual plants[8]).

The Bayesian coalescent based methods implemented in the computer program BEAST[29] are ideally suited to inferring nucleotide substitution rates from temporally structured datasets such as ours. Applying these methods we estimated mean substitution rates of approximately 3.5 × 10^-4^, 2.0 × 10^-4 ^and 2.1 × 10^-4 ^sub/site/year over the duration of the SSRV, MSV-F and MSV-B experiments, respectively (Figure 2). These estimates were reasonably consistent irrespective of the molecular clock or demographic models used. All had overlapping 95% highest probability density (HPD) intervals within the range of 7.22 × 10^-5 ^(observed with the MSV-F dataset using a relaxed clock + Bayesian skyline plot model) to 6.77 × 10^-4 ^subs/site/year (observed with the SSRV dataset using a relaxed clock + Bayesian skyline plot model; Figure 2).

These rates are slightly lower than those of ~7 × 10^-4 ^subs/site/year previously estimated for MSV-A, MSV-B and MSV-C in one- to five-year long evolution experiments involving cloned virus genomes [9]. They are, however, approximately equivalent to those estimated within a natural temporally-structured tomato infecting begomovirus dataset employing the same methodology used here (Figure 2; [4]). Our results in relation to these other studies are entirely unsurprising: it is expected that substitution rate estimates from shorter term evolution experiments will be closer to the basal mutation rate than those estimated either from longer term experiments, or from natural sequences sampled over a number of decades [15].

**Figure 3:**
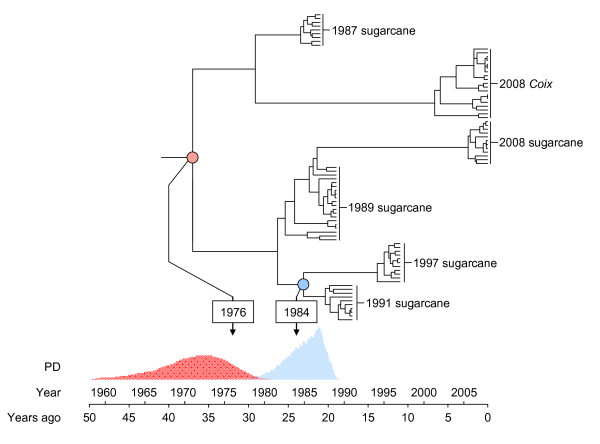
**The maximum clade credibility phylogenetic tree recovered under one of the best-fit models (exponential growth strict-clock) identified using BEAST Almost identical results were obtained under the constant population size strict-clock model (available from the authors on request)**. The best fit model indicates that: (1) the sugarcane-to-*Coix *SSRV transmission event that initiated the experiment, which actually occurred in 1976, was estimated to have occurred in 1971 (95% highest clade credibility interval = 1962–1979, indicated by the red posterior probability distribution beneath the tree) and (2) the date of the three-way 1984 sugarcane virus population split was estimated to have occurred in 1985 (95% highest probability density = 1980 – 1989 indicated by the blue posterior probability distribution for the tMRCA situated beneath the tree). Thus, applying the estimated SSRV substitution rate quite accurately recovers the dates of two important events in the 32-year long SSRV evolution experiment.

Importantly, the structure of the SSRV experiment allowed us to verify the accuracy of our SSRV nucleotide substitution rate estimate. Firstly, we knew that the date associated with root node separating the 2008 *Coix *samples from the 1989, 1991, 1997 and 2008 sugarcane samples was 1976 – the year in which viruses were transmitted from sugarcane to *Coix*. Secondly, we knew that in 1984 two lineages represented by the 1991 and 1997 sugarcane samples were split from the lineage represented by the 1989 and 2008 samples (Figure 3).

Irrespective of the demographic and clock models used, the mean estimated date of the 1984 sugarcane lineage split was within 4 years of the actual date, and the estimated mean date of the sugarcane to *Coix *transmission event was within 8 years of the actual date. In all cases the 95% HPD intervals included the actual dates (Figure 3). The constant size and exponential growth strict-clock models provided a significantly better fit to the data than the relaxed-clock models while the opposite pattern was observed for the Bayesian skyline plot model (see additional file 1). The exponential growth and constant population size strict molecular clock models both fitted the data equally well however, with the former recovering a marginally higher likelihood than the latter model. These models yielded more accurate estimates of the 1976 sugarcane to *Coix *transmission event and the 1984 sugarcane lineage split (within five and one years of the actual dates, respectively), as well as narrower 95% HPD intervals.

These fairly-precise recapitulations of a known bifurcation and a known trifurcation in our experiment serve as independent confirmation that, at the very least, our substitution rate estimates for SSRV using the strict-clock model (between 2.27 × 10^-4 ^and 2.86 × 10^-4 ^subs/site/year) were reasonably accurate irrespective of the demographic models used.

The SSRV results are the first substitution rate estimates from a plant virus maintained in laboratory/greenhouse settings that allowed the same heterochronous sampling over the tens of years that are used to estimate rates from field-isolated viruses. The agreement between the laboratory substitution rate of a mastrevirus and the field substitution rate of begomoviruses (Figure 2) indicates that the different, potentially relaxed, selection pressures viruses face in greenhouse-maintained plants do not lead to different rates of evolution.

### Specific nucleotide substitution biases are conserved across the geminiviruses

Analyses of virus genome sequences both sampled from nature and in controlled evolution experiments have indicated that higher than expected geminivirus mutation rates are at least partially attributable to the susceptibility of ssDNA to oxidative damage [4,5,9]. The signatures of such damage are elevated rates of C → T, G → A and G → T mutations. Whereas ssDNA is known to be more prone than dsDNA to the oxidative deamination reactions that cause C → T and G → A transitions [30-32], it is also more prone to reactions that convert guanine to 8-oxoguanine and cause G → T transversions [33-35].

**Figure 4:**
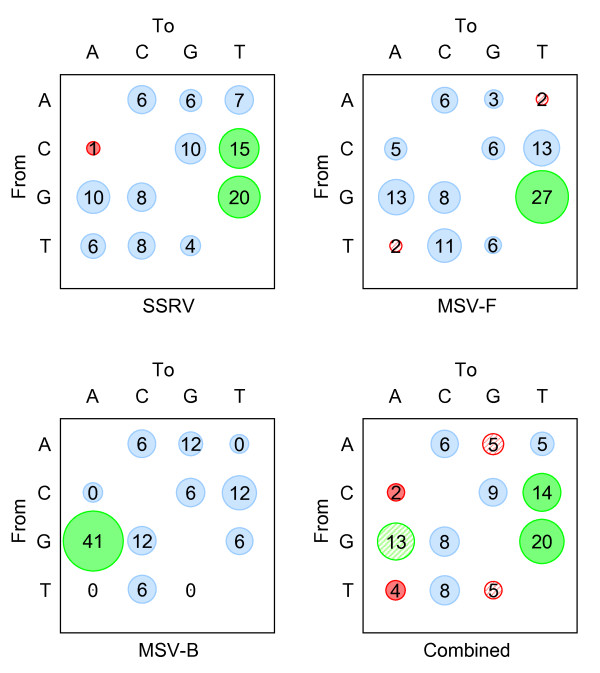
**Inferred numbers of substitutions for each pair of nucleotides as determined through reconstructing ancestral sequences under the non-reversible (12 rate) maximum likelihood model**. Sizes of circles are proportional to relative nucleotide substitution rates, whereas counts are inferred numbers of substitutions along the phylogeny, given the maximum likelihood model (expressed as a percentage of the total number of inferred mutations). Counts were used for Chi-square tests (described in methods). Given the expectation that all mutation types are equally likely, circles are colored blue when the mutations they represent are neither more nor less common than expected, red when they are less common than expected and green when they are more common than expected. The hatched circles indicates that although transitions and transversions are are respectively more or less common than would be expected if all mutation types were equally probable, if one only considers the frequencies of transitions in relation to other transitions and transversions in relation to other transversions, then these, mutations are no more or less common than expected.

In each of the three independent evolution experiments, we estimated the relative non-reversible rates of substitution between nucleotides (e.g. the rate of A → C is not necessarily the same rate as C → A) using a maximum likelihood approach implemented in the program HYPHY[36]. In both the SSRV and MSV-F experiments, C → T, G → A and G → T substitutions were inferred to have higher relative rates than all nine other substitution types (Figure 4). Although C → T and G → A transitions also had the highest relative rates in the MSV-B experiment, in this experiment G → T transversions had only the seventh highest rate. It is important to point out, however, that there were only 17 polymorphisms in the entire MSV-B dataset. Since the SSRV and MSV-F datasets respectively contained 157 and 64 polymorphisms, their relative substitution rates may be more meaningful.

To determine whether specific types of mutation occur more or less frequently during MSV and SSRV evolution than could be accounted for by chance, we collectively considered all 238 mutations observed to have occurred during our three evolution experiments using the chi square test outlined by van der Walt *et al*. [9]. This analysis revealed that whereas C → T, G → A and G → T mutations were indeed significantly over-represented (chi square p = 4 × 10^-4^, 7 × 10^-3^, and < 1 × 10^-5^, respectively), C → A, T → A and T → G transversions were significantly under-represented (chi square p = 7 × 10^-3^, 2 × 10^-2 ^and < 4 × 10^-3 ^; Figure 4).

All four possible transition mutations, including C → T and G → A, are generally thought to occur at higher frequencies than the eight possible transversion mutations [37]. Indeed, our results across all the evolution experiments indicate individual transition substitutions occurred at approximately twice the frequency of individual transversion substitutions (Figure 4). Accordingly, when we restricted our chi square test to include only either transitions or transversions the frequency of G → A mutations was no longer significantly higher than that of the other transition mutations. Similarly, whereas the frequency of T → G mutations was not significantly lower than those of other transversion mutations, the frequency of A → G mutations was inferred to be significantly lower than those of other transition mutations. However, the C → T and G → T substitutions remained significantly higher than expected and the frequencies of the C → A and T → A substitutions still lower than expected.

Despite the relatively good agreement of overrepresented substitutions between begomovirus studies [4,5] and our evolution experiments, there isn't perfect concordance among substitution biases in different geminiviruses. For example, whereas both our study and a Tomato yellow leaf curl virus (TYLCV) study indicate that T → G substitutions are significantly underrepresented during the evolution of some geminiviruses, this type of substitution has been significantly over-represented during East African cassava mosaic virus evolution [5].

### Substitution biases are strand specific

As only the virion strands of geminivirus genomes spend significant time in a single stranded state, an additional signature that would indicate that ssDNA is more prone than dsDNA to mutation should be the existence of strand specific substitution biases. While the overrepresented C → T and G → A transitions are likely occurring on the virion strand, these two transitions are complementary and cannot be used to determine strand-specificity. However, G → T substitutions occur at a higher frequency than C → A substitutions (i.e. the complement of G → T) providing clear evidence either that: (1) C → A mutations occur much more frequently on the complementary strand than they do on the virion strand; or (2) G → T mutations occur much more frequently on the virion strand than they do on the complementary strand. It is possible to choose between these two alternatives if, as is the case with geminiviruses, only one strand spends an appreciable amount of time in a single-stranded state.

We devised a likelihood ratio test to determine whether there was significant evidence of a strand-specific substitution bias in our three evolution experiments. This simply involved determining the relative likelihoods of observing our data given either (1) a six rate substitution matrix in which complementary mutations were constrained to occur at the same rate (i.e. a situation with no strand specific substitution biases) or (2) a twelve rate substitution matrix in which all substitution types were free to occur at different rates.

For both the SSRV and MSV-F experiments this test inferred the existence of significant strand specific nucleotide substitution biases (chi square p = 8.5 × 10^-3 ^and 5.7 × 10^-4 ^respectively) strongly indicative of mutational processes operating specifically on ssDNA. Possibly because of the low numbers of polymorphisms considered, the test failed to reveal any such evidence for the MSV-B dataset.

Such strand specific substitution biases taken together with increased rates of specific substitutions such as G → T, C → T and G → A amongst both mastrevirus and begomovirus datasets indicate very strongly that (1) all geminiviruses probably experience roughly equivalent mutagenic stresses and (2) high geminivirus substitution rates are, in part, driven by shared mutagenic processes independent of polymerase error, operating on ssDNA.

### Negative and positive selection against a background of neutral genetic drift

The co-divergence hypothesis of Wu *et al*. [23] demands that, over thousands of years, at least 99.999% of all arising mutations and 99.99% of all substitutions that appear dominant in populations over tens of years are ultimately purged from mastrevirus populations by negative selection. Although it is impossible to directly test this hypothesis by running controlled evolution experiments over such long time-periods, it is possible to directly test this supposition by looking for the predicted signal of overwhelming negative selection in our evolution experiments.

In our SSRV evolution experiment we detected significant evidence (p < 0.1) of negative selection operating on 12 of the 22 *cp *and 10 of the 48 *rep *codons displaying some degree of nucleotide variation (Table 2). This indicated that there is not strong purifying selection purging 99.999% of nucleotide variation, and implies that at least some mastrevirus nucleotide variation is selectively neutral. It is important to note that Wu *et al*. [23] themselves did not find any evidence for stronger purifying selection, as determined by the ratio of non-synonymous to synonymous substitutions, among their WDV isolates than have virologists who argue for fast long-term evolution in geminiviruses [4,5]. Of course, these ratios only quantify negative selection acting on expressed amino acid sequences – not negative selection acting directly on the underlying nucleotide sequences. Even Wu *et al*. [23] are tacitly accepting that large numbers of synonymous nucleotide substitutions are probably selectively neutral, weakening their argument that negative selection on all genetic change is overwhelming and efficient. Importantly, we also detected two codons in *mp *and one in *rep *that are apparently evolving under positive selection (posterior probability ≥ 0.99; Table 2). It is very difficult to reconcile the extremely strong negative selection demanded by the co-divergence hypothesis with this demonstration that natural selection does not even uniformly disfavour non-synonymous mutations.

**Table 2:** Site-by-site signals of positive and negative selection acting on movement protein (*mp*), coat protein (*cp*) and replication associated protein (*rep*) gene codons during the SSRV evolution experiment

Gene	Codon	Method^*a*^	Selection^*b*^	Motif/domain (site underlined where relevant)
*mp*	21	R	+	
	63	R	+	C-terminal boundary of hydrophobic domain

*cp*	3	R	-	DNA Binding domain
	67	R	-	DNA Binding domain
	69	R	-	DNA Binding domain
	85	FR	-	DNA Binding domain
	105	FR	-	DNA Binding domain
	122	FR	-	
	136	R	-	
	157	FRS	-	
	180	R	-	
	201	R	-	
	217	R	-	
	219	R	-	

*rep*^*b*^	7	FR	-	
	28	FR	-	RCR motif I (FLTYPHC)
	30	FR	+	
	133	FR	-	
	147	FR	-	
	155	FR	-	
	158	FR	-	
	185	FRS	-	Rep-Rep oligomerisation domain (ASKLFPDTVEEY)
	321	FR	-	
	326	FR	-	
	356	FRS	-	

^*a *^F = Fixed effects likelihood method; R = Relative effects likelihood method; S = Single likelihood ancestor counting method.

^*b *^+ = evidence of positive selection (p-value < 0.1); - = evidence of negative selection (p-value < 0.1).

^*c *^Excludes codons 217–282 that are expressed in different frames in *rep *and *repA*.

In fact, the degree of negative selection implied by the co-divergence hypothesis would be expected to produce a situation in which all mutants would only be detectable for a short period of time after they arise – thereafter they would be expected to become extinct due to their inability to compete effectively with wild-type viruses. Under such conditions the overwhelming majority of detectable mutations should be unique to the mutant genomes that carry them. This pattern of genetic variation is generally detected using population genetic neutrality tests such as Tajima's D [38] or Fu and Li's F* statistics [39] that describe the representation in datasets of mutations that are found only in individual sequences relative to those that are found in multiple sequences. If these statistics have a significantly negative value for a group of sequences randomly sampled from a population of constant size, it implies that the accumulation of mutations within the sequences was more strongly influenced by negative selection than it was by neutral genetic drift.

We were unable to find any significant deviation from zero for either Tajima's D or Fu and Li's F* statistics in any of the virus populations we sampled during our evolution experiments (Table 1). Although negative scores for both these statistics for most of the populations imply that sequences were subjected to some degree of negative selection, it is apparent that random genetic drift is the dominant process determining the relative frequencies of particular mutations in these populations. For example, although only one sequence differed from all the rest at 53 out of 128 variable nucleotide sites in the SSRV dataset, the remainder were sites at which mutations were present in multiple sequences and were therefore not significantly deleterious.

From our evolution experiment data it is very simple to directly infer the action of genetic drift and/or positive selection acting on mutations by tracking changes in the population-wide frequency of particular mutants over time. For example, in the SSRV experiment, we observed 8 instances where mutations that were present in <25% of sequences sampled in 1989, were present in 100% of sequences sampled from the same plant in 2008 – these mutations could only have reached fixation by 2008 through either genetic drift or positive selection. Taken collectively, all our data clearly indicate the mutations that arose during our controlled evolution experiments were not uniformly subject to anywhere near the degree of negative selection required by the co-divergence hypothesis.

### Congruent phylogenies are necessary, but not sufficient, to demonstrate virus-host coevolution

As has been pointed out by the originators of the mastrevirus-host co-divergence hypothesis, it very difficult to prove virus-host co-speciation [23,40]. For example, it is usually impossible to confirm that phylogenetic signals superficially indicative of co-divergence are not instead caused by other epidemiological and ecological factors [see [40] for specific examples of how these can be confused with co-divergence]. Mismatched substitution rates between viruses and their hosts have provided evidence against some long-assumed co-divergence pairs, including hantaviruses and their rodent hosts [41] and JC virus, whose phylogeny had been used as a proxy for early human migration patterns [42]. For example, the close relationships between Human immunodeficiency virus and other closely related lentiviruses isolated from simians are also superficially indicative of co-divergence. Despite this it is now clear that the apparent correspondence of such virus and host relationships is as a result of viruses being more capable of adapting to new host species if the new host species are genetically similar to their old host species [40]. The ability of geminiviruses to adapt rapidly to novel hosts, and the polyphagy of their insect vectors also argue both against the hypothesis of widespread co-speciation among these viruses and in favour of the hypothesis that apparent co-speciation signals simply reflect the fact that genetically more similar viruses just happen to infect, and become specifically adapted to, genetically more similar hosts. The balance of evidence therefore still strongly favours geminiviruses having RNA-virus-like substitution rates that exclude the possibility of their having co-diverged with their hosts.

## Conclusion

We have used long-term evolution experiments to investigate the credibility of recent suggestions that mastreviruses may have co-diverged with their host species over millions of years. We have shown that both the mutational processes and the substitution rates they drive are conserved across the geminivirus family, and are orders of magnitude higher than the rates implied by the co-divergence hypothesis. Additionally, we have provided evidence against potent negative selection as a plausible mechanism by which very-long-term mastrevirus substitution rates could be more than 10,000 fold lower than both their basal mutation rates and directly measured substitution rates. While some of the genetic variation in our three evolution experiments is under statistically significant positive selection, much of it appears nearly neutral. In short, all available evidence suggests that mastrevirus evolution is no more severely constrained by negative selection than is that of other rapidly evolving viruses [15].

## Methods

### Virus isolates

A sugarcane plant presenting with streak symptoms was collected in 1971 from a multiplication plot at Médine, Mauritius, and was used in 1976 as a source of inoculum to infect both a sugarcane plant (variety H44-3098) and a *Coix lacryma-jobi *plant. These were maintained in an insect free glass house for the next 32 years at the Mauritius Sugar Industry Research Institute. Virus was retransmitted from the *Coix *plant to a second sugarcane plant at some time between 1977 and 1986. Samples were taken from the original H44-3098 plant in 1989 and 2008; from the second sugarcane plant in 1987; and from the *Coix *plant in 2008. In 1984 two separate cuttings from the H44-3098 plant were taken and maintained separately – samples were taken from one of these cuttings in 1991 and from the other in 1997.

Two *Digitaria *plants with mild streak-like symptoms were collected in Rwanda and Burundi by R.H. Markham (the then plant pathologist at the CAB International Institute of Biological Control, Kenya) in 1984. After transferring them to the John Innes Centre in Norwich, UK, viruses were leafhopper transmitted from these plants to *Digitaria sanguinalis*. These two newly infected *D. sanguinalis *plants were maintained under insect free conditions between 1984 and 1997 with samples being taken from each plant in both 1991 and 1997.

### Isolation, cloning and sequencing of viral DNA

Total DNA was isolated from preserved sugarcane or *Digitaria *samples by either a modified CTAB method [43,44] or the Extract-N-Amp™ Plant (Sigma-Aldrich) method as described by Shepherd *et al*. [45]. The virus was amplified using phi29 DNA polymerase (TempliPhi™, GE Healthcare, USA; [46]), the amplified concatemers were digested with *Sal*I (sugarcane virus isolates) or *Bam*HI (*Digitaria *virus isolates) to yield ~2.7-kb linearised viral genomes which were cloned into pGEM3Zf+ (Promega Biotech) cloning vector. Both strands of cloned genomes were commercially sequenced (Macrogen Inc., Korea) by primer walking. Sequences were assembled and edited using DNAMAN (version 5.2.9; Lynnon Biosoft) and MEGA (version 4 [47]).

### Detection of recombination and phylogenetic tree construction

Sequences from all three evolution experiments were tested for evidence of recombination using LDHAT[48] and various methods implemented in the program RDP3[49]. These analyses failed to detect any significant evidence of recombination in our datasets. Phylogenetic trees were constructed using PHYML[50] with best fit models automatically selected by RDP3[49].

### Estimation of nucleotide substitution rates

A co-estimate of the nucleotide substitution model parameters, phylogeny and time to the most recent common ancestor (tMRCA) was obtained for the MSV-B, MSV-F and SSRV datasets using the Bayesian Markov chain Monte Carlo (MCMC) method implemented in BEAST v1.4.8 [29]. Six different coalescent demographic models were employed including both parametric (constant population size, exponential population growth) and non-parametric (Bayesian skyline plot; BSP) models, with both a strict and relaxed (uncorrelated LogNormal prior) molecular clock.

For each evolutionary model, two independent runs of length 5 × 10^7 ^steps in the Markov chain were performed using BEAST and checked for convergence using TRACER v1.4 [29]. The estimated sample sizes for each run were almost always > 200 indicating sufficient mixing of the Markov chain and parameter sampling. When similar results were produced from independent runs of the Markov chain, the log files were combined with the program LOGCOMBINER v1.4.7 available in the BEAST package [29].

### Demographic and clock model comparisons

Models were compared by calculating a measure known as the Bayes factor, which is the ratio of the marginal likelihoods of the two models being compared [51,52]. Bayes factors allow the comparison of non-nested models (such as the non-parametric Bayesian skyline plot vs. the parametric constant or exponential growth demographic models) that cannot be validly compared using the mean log posterior probabilities.

### Analysis of nucleotide substitution biases

We evaluated nucleotide substitution biases using maximum likelihood phylogenetic models of evolution. Briefly, such models make use of a continuous time Markov process in which mutations are modelled along branches of a phylogenetic tree, according to a rate matrix with elements (*q*_*ij*_) describing the instantaneous substitution rate from nucleotide *i *to nucleotide *j*. These elements (*q*_*ij*_) typically include (i) parameters describing equilibrium nucleotide frequencies, (ii) exchangeability parameters describing the nucleotide substitution process and (iii) rate heterogeneity parameters accounting for spatial heterogeneity of the substitution process [53]. The most general form of the nucleotide substitution matrix, or general time reversible model (GTR; [54]), has elements



where *π*_*j *_is the equilibrium frequency of nucleotide *j *assumed to be in equilibrium and constant across lineages; and *θ*_*ij *_the instantaneous rate of substitution of nucleotide *i *with nucleotide *j*. These models typically assume time-reversibility such that *θ*_*ij *_= *θ*_*ij*_. Here we use standard model comparison techniques to compare reversible with non-reversible models of evolution as applied to mastreviruses. We implemented a standard GTR model (where forward and reverse substitutions are constrained to have the same rate, for example, C → T substitution rates must be the same as T → C substitution rates) and a different non-reversible model of evolution with six rates, in which rates are shared by complementary substitutions (e.g. C → T rates are constrained to be the same as G → A rates). Both six rate models are nested within the non-reversible twelve-rate model (where all 12 substitutions are free to occur at different rates), and thus a likelihood ratio test with degrees of freedom equal to the difference in the number of parameters is appropriate for model comparisons between each of the six rate models and the 12 rate model. Phylogenetic models and statistical tests were implemented in the HYPHY batch language [36] and are available from the authors on request.

We reconstructed ancestors at internal nodes using maximum likelihood and a non-reversible substitution model, and counted substitutions along branches of the phylogeny using HYPHY[36]. The relative counts of each mutation type over the 32 years of the SSRV experiment and the 6 years of the MSV-B and MSV-F experiments were compared using the 2 × 2 chi square test described by van der Walt *et al*. [9]. This takes into account nucleotide composition biases but not inherent differences in rates of transition vs. transversion mutations. We therefore also used a modified version of this test where transitions and transversions were treated separately such that, for example, the number of times that a particular transversion mutation was estimated to have occurred was only compared to the collective number of times that the seven other transversion mutation types were estimated to have occurred.

### Site by site analysis of natural selection

We used three methods implemented on the DATAMONKEY webserver [55] that examine ratios of non-synonymous (*dN*) and synonymous mutations (*dS*) to identify signals of positive (dN > dS) and negative selection (*dN *<*dS*) operating on individual codons within genes. Single likelihood ancestor counting (SLAC) infers selection by comparing observed rates of non-synonymous and synonymous mutation at each codon to that expected under a binomial distribution (SLAC). Fixed effects likelihood (FEL) compares model fit in which non-synonymous and synonymous mutations are constrained to be equal, to an unconstrained model (FEL). Random Effects Likelihood (REL) methods approximate the distribution of non-synonymous to synonymous rates across all sites into classes, and calculate the posterior probability that each site belongs to each of the rate classes. Since these methods perform better on larger data sets [56] we only conducted these analyses on sequences obtained during the SSRV experiment. We tested alignments of genes for the movement protein (*mp*, 79 sequences, 327 nucleotides long), coat protein (*cp*, 78 sequences, 741 nucleotides long) and the replication-associated protein (*rep*, 80 sequences, 888 nucleotides long, excluding the alternate reading frame overlap between *rep *and *repA *codons 217–282). The number of sequences varied between alignments because we excluded sequences with apparent indels or premature stop codons.

### Neutrality tests

Tajima's D and Fu and Li's F* statistics [38,39] were calculated and tested for significance using the program DNASP version 4.0 [57]. Between 8 and 20 full length genomes randomly cloned from each of the six SSRV samples and the 2 MSV-B and MSV-F samples were tested. All the samples from each of the SSRV, MSV-B and MSV-F experiments were also analysed together. Both D and F* statistics identify the contribution of rare variants to total genetic diversity. Significantly negative statistics are indicative of an excess of rare variants and are a signature of very strong negative selection against the survival of mutant genomes [38,39].

## Abbreviations

BF: Bayes factor; BSP: Bayesian skyline plot; CP: coat protein; *cp*: coat protein gene; EACMV: East African cassava mosaic virus; GTR: general time reversible; HPD: highest probability density; MCMC: Markov chain Monte Carlo; MP: movement protein; *mp*: movement protein gene; MSV: Maize streak virus; Rep: replication associated protein; *rep*: replication associated protein gene; ssDNA: single stranded DNA; SSRV: Sugarcane streak Réunion virus; tMRCA: time to the most recent common ancestor; TYLCCV: Tomato yellow leaf curl China virus; TYLCV: Tomato yellow leaf curl virus; WDV: Wheat dwarf virus.

## Competing interests

The authors declare that they have no competing interests.

## Authors' contributions

SS, AV and DPM conceived the study; AV, ALM, BEO DNS and LD cloned and sequenced the virus genomes; WD devised the nucleotide substitution bias test; GH, NW and SD carried out the nucleotide substitution rate analyses; SS and GT initiated the sugarcane/*Coix *experiment and maintained the plants for over 32 years; RWB and EPR provided archived samples for analysis; SD and DPM carried out the selection analyses; DPM carried out the recombination analysis; AV, GH, SD, WD and DPM prepared the manuscript. All authors other than PGM read and approved the final manuscript.

## Supplementary Material

Additional file 1 Comparisons of the Bayes factors between different evolutionary models for the MSV-B, MSV-F and the SSRV datasets. Click here for file
