# Supplementary material for: Experimental evidence indicating that mastreviruses probably did not co-diverge with their hosts
Source: Virol J. 2009 Jul 16;6:104. doi: 10.1186/1743-422X-6-104 (PMC2719613; doi:10.1186/1743-422X-6-104)
Supplement: Additional file 1 — Comparisons of the Bayes factors between different evolutionary models for the MSV-B, MSV-F and the SSRV datasets. [file 1743-422X-6-104-S1.doc]

Additional file 1. Comparisons of the Bayes factors between different evolutionary models for the MSV-B, MSV-F and the SSRV datasets.

| Model Comparisons | MSV-B | | MSV-F | | SSRV | |
| --- | --- | --- | --- | --- | --- | --- |
|  | 2ln BF | Evidence against Ho | 2ln BF | Evidence against Ho | 2ln BF | Evidence against Ho |
| Const Strict (H0) vs Relaxed (H1) clock | 3.94 | Positive | 7.79 | Strong | -37.12 | None |
| Expo Strict (H0) vs Relaxed (H1) clock | 3.92 | Positive | 8.21 | Strong | -37.71 | None |
| BSP strict (H0) vs Relaxed (H1) clock | 2.59 | Weak | 8.66 | Strong | 13.48 | Strong |
| Const (H0) vs Expo (H1) Relaxed clock | 0.86 | Weak | 0.10 | Weak |  |  |
| Const (H0) vs Expo (H1) Strict clock |  |  |  |  | 0.08 | Weak |
| Const (H0) vs BSP (H1) Relaxed clock | 2.30 | Weak | 0.05 | Weak |  |  |
| BSP (H0) vs Expo (H1) Relaxed clock | 1.44 | Weak | 0.15 | Weak |  |  |
| BSP (H0) vs Constant (H1) Strict clock |  |  |  |  | 49.97 | Strong |
| BSP (H0) vs Exponential (H1) Strict clock |  |  |  |  | 49.90 | Strong |

BF = Bayes factor - the difference (in log space) of the marginal likelihood of the null (H0) and the alternative (H1) hypotheses. The Bayes factors were estimated by comparing the approximate marginal likelihoods of the different models given in Additional file 2.
